# The reference ranges and characteristics of lymphocyte parameters and the correlation between lymphocyte parameters and routine health indicators in adults from China

**DOI:** 10.1186/s12979-022-00298-5

**Published:** 2022-09-27

**Authors:** Wei Liu, Jie Xu, Qiyao Pu, Mingwei Lan, Xiaolu Zhang, Yufeng Gu, Ying Wang, Fan Zheng, Jingjing Qian, Chunxue Fan, Jun Sui, Yanli Xu, Yongchen Zhang, Jing Luo, Xiangyang Lin, Shaorui Shi, Liying Wang, Chengming Sun, Maohua Zhou, Baohong Yue, Feng Wang

**Affiliations:** 1 Department of Laboratory Medicine, Tongji Hospital, Tongji Medical College, Huazhong University of Science and Technology, Wuhan, China; 2 Department of Neurology, Beijing Tiantan Hospital, Capital Medical University, Beijing, China; 3 Department of Laboratory Medicine, The First Affiliated Hospital of Zhengzhou University, Zhengzhou, China; 4 Department of Laboratory Medicine, Guangdong Provincal People’s Hospital, Guangdong Academy of Medical Sciences, Guangzhou, China; 5 Department of Clinical Laboratory, Yantai Yuhuangding Hospital of Qingdao University, Yantai, China; 6 Institute of Pediatrics, The First Hospital of Jilin University, Changchun, China; 7 Department of Clinical Laboratory, The Second People’s Hospital of Yibin West ChinaYibin Hospital, Sichuan University, Yibin, China; 8 Department of Clinical Laboratory, The First Affiliated Hospital of Wenzhou Medical University, Wenzhou, China; 9 Division of Rheumatology, Department of Medicine, The Second Hospital of Shanxi Medical University, Taiyuan, China; 10 Department of Laboratory Medicine, the Second Hospital of Nanjing, Nanjing University of Chinese Medicine, Nanjing, China; 11 Department of Hematology Laboratory, Guangzhou First People’s Hospital, South China University of Technology, Guangzhou, China

**Keywords:** Immune function, Lymphocyte parameters, Current function, Lymphocyte potential, Nutritional indicators, Lipid profile

## Abstract

**Background:**

Assessment of immune function is of key importance in recognition of disease or healthy status, which still faces challenge in clinical practice. We conducted a 10-center study to investigate lymphocyte parameters including the number, phenotype and IFN-γ-producing ability, and routine laboratory indicators by using the standard method.

**Results:**

Although the heterogeneity of lymphocyte parameters was widely found, we have established the normal ranges of these parameters by using pooled data which showed no significant difference among centers. Cluster analysis of 35 parameters found 3 interesting clusters which represented different immunological status. Cluster 1 (parameters: IFN-γ^+^CD4^+^ T cell percentage and IFN-γ^+^CD8^+^ T cell percentage) represented current lymphocyte function, which was associated with indicators such as body mass index and red blood cell; Cluster 2 (parameters: NK cell number and CD45RA^+^CD4^+^ T cell percentage) represented potential of lymphocytes, which was associated with indicators such as albumin and high-density lipoprotein. Cluster 3 (parameters: HLA-DR^+^CD8^+^ T cell percentage) represented inflammatory status, which was associated with indicators such as low-density lipoprotein, globulin and age. Correlation analysis found that nutritional indicator albumin is significantly positively correlated with lymphocyte potential. Triglyceride and body mass index were positively correlated with current lymphocyte function rather than lymphocyte potential. The loss of CD8^+^ T cells was extremely pronounced with increasing age and was one of the most important factors to cause immunosenescence, which may be associated with increased glucose.

**Conclusions:**

We have established the normal ranges of lymphocyte parameters in different areas. This study elucidates the key indicators used to reflect the current function or potential of lymphocytes, which may provide a valuable clue for how to keep immunity healthy.

**Supplementary Information:**

The online version contains supplementary material available at 10.1186/s12979-022-00298-5.

## Background

Assessment of immune function is of key importance in primary recognition of disease or healthy status. Lymphocytes, the most important cells in immune system, are composed of T, B and natural killer (NK) cells, which can be used to reflect the cellular, humoral and innate immunity [1]. Quantification of these lymphocytes provides clinical help in the diagnosis, treatment as well as prognosis of diseases such as immunodeficiency and autoimmune disease, infection, and cancer [2–9], and is also an effective way to define health and immunosenescence [10–14]. Despite the importance of lymphocyte subset quantification, additional analysis of the phenotype and function of lymphocytes is necessary for accurate evaluation of immunity. Recently, the detection of IFN-γ-producing ability after stimulation are related in the expected ways to define the current state of lymphocytes [15, 16]. The number of T cells, especially CD45RA^+^ and CD28^+^ T cells, can be used as the symbol of the potential of lymphocytes [17–19]. Besides, increased HLA-DR expression on T cells correlates with more severe inflammation [20–22]. Generally, increasing evidences support the notion that combination detection of the number and function of lymphocytes becomes a practical way to evaluate immunity.

However, the greatest dilemma in the field is the heterogeneity of peripheral lymphocytes, appeared in both composition and function. It is now clear that the heterogeneity of lymphocyte subset quantification is not only related to diseases and medications but also to many factors such as geographical location, age, gender, ethnicity, stress, physical activity, lifestyle, and even circadian rhythms [23–26]. Besides, the variation of methods such as sample preparation and flow cytometer operation also induce biological variability across laboratories [27]. These data emphasize the use of domestic reference values is necessary to improve the accuracy of lymphocyte data interpretation.

Although the reference ranges of both number and phenotype of lymphocyte subsets have been widely studied in different countries including China [28–33], there are rare studies to investigate lymphocyte parameters considering population heterogeneity, comprehensiveness of indicators, and method variation simultaneously. In addition, no study has focused on systematically characterizing the relationship between lymphocyte parameters and routine health indicators. In view of these shortcomings, we conducted a 10-center study to investigate lymphocyte number, phenotype and IFN-γ-producing ability by using the standard method, and also to compare these lymphocyte parameters with routine indicators. To the best of our knowledge, this is the first study to report the reference ranges of comprehensive lymphocyte parameters in most geographical areas of China, and we have also elucidated the routine laboratory indicators which could be used to reflect the current function or the potential of lymphocytes.

## Methods

### Study population

**Fig. 1:**
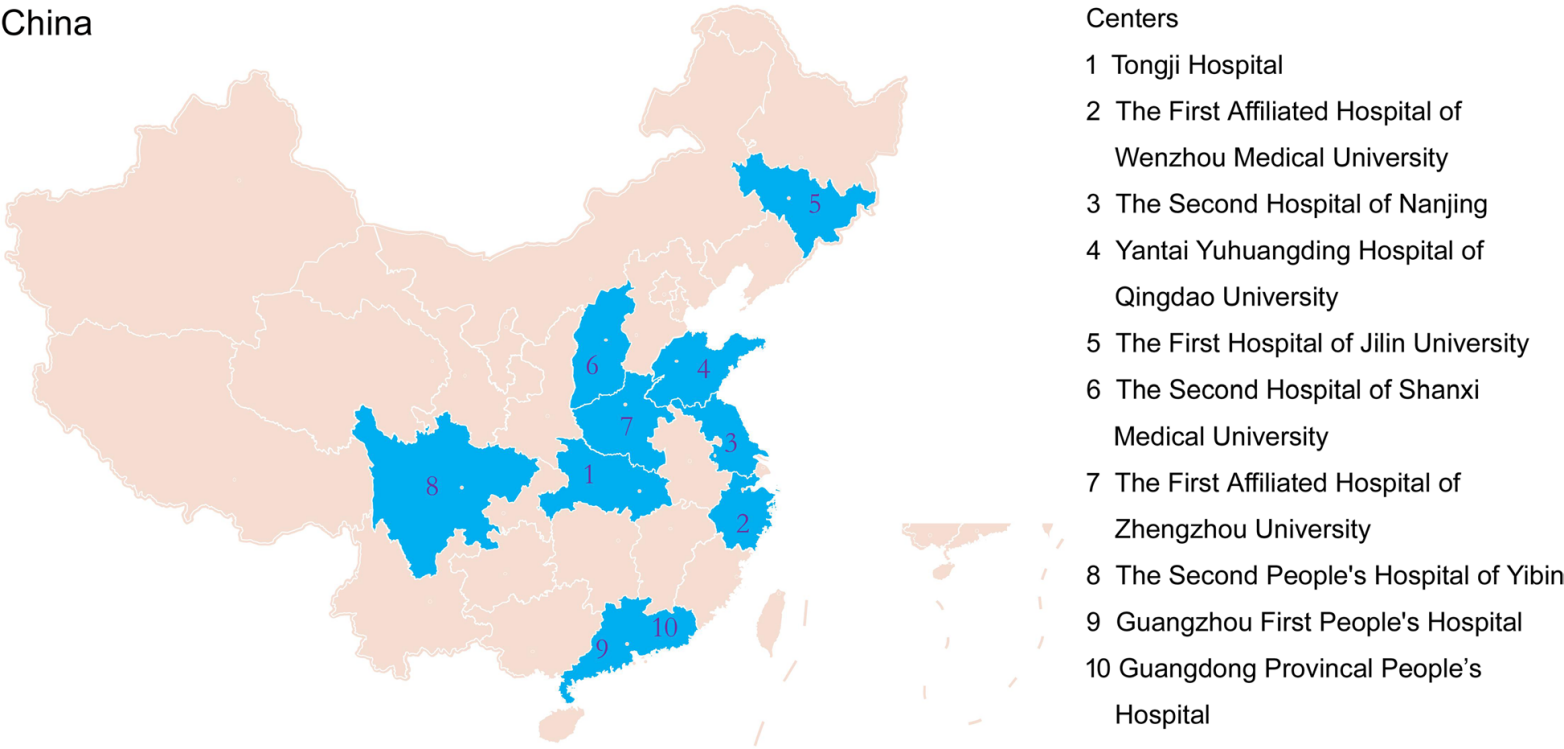
Geographical location of 10 hospitals distributed in 9 provinces in China

This is a multicenter descriptive study. Between February 2020 and July 2020, peripheral blood from healthy individuals aged from 20 to 60 years were collected from 10 university-affiliated tertiary hospitals, which were located in the eastern, western, southern, northern, and central regions of China (Fig. 1). Healthy individuals were defined as having no clinical and radiographic evidences of active diseases. Exclusion criteria were as follows: pregnancy or breast-feeding, hematological disease, cancer, and never receiving immunosuppressants or other similar agents. The demographic and physiological characteristics of the participants were collected from electronic health management information system. This study was approved by the Ethics Committee of Tongji Hospital, Tongji Medical College, Huazhong University of Science and Technology; the Ethics Committee of The First Affiliated Hospital of Zhengzhou University; the Ethics Committee of Guangdong Provincal People’s Hospital, Guangdong Academy of Medical Sciences; the Ethics Committee of Yantai Yuhuangding Hospital of Qingdao University; the Ethics Committee of The First Hospital of Jilin University; the Ethics Committee of The Second People's Hospital of Yibin West China Yibin Hospital; the Ethics Committee of The First Affiliated Hospital of Wenzhou Medical University; the Ethics Committee of The Second Hospital of Shanxi Medical University; the Ethics Committee of the Second Hospital of Nanjing, Nanjing University of Chinese Medicine; and the Ethics Committee of Guangzhou First People's Hospital, South China University of Technology, China. Requirement for written informed consent was waived by the institutional ethics board because all measurements were performed in residual blood samples.

### Sample preparation and flow cytometric analysis

**Fig. 2:**
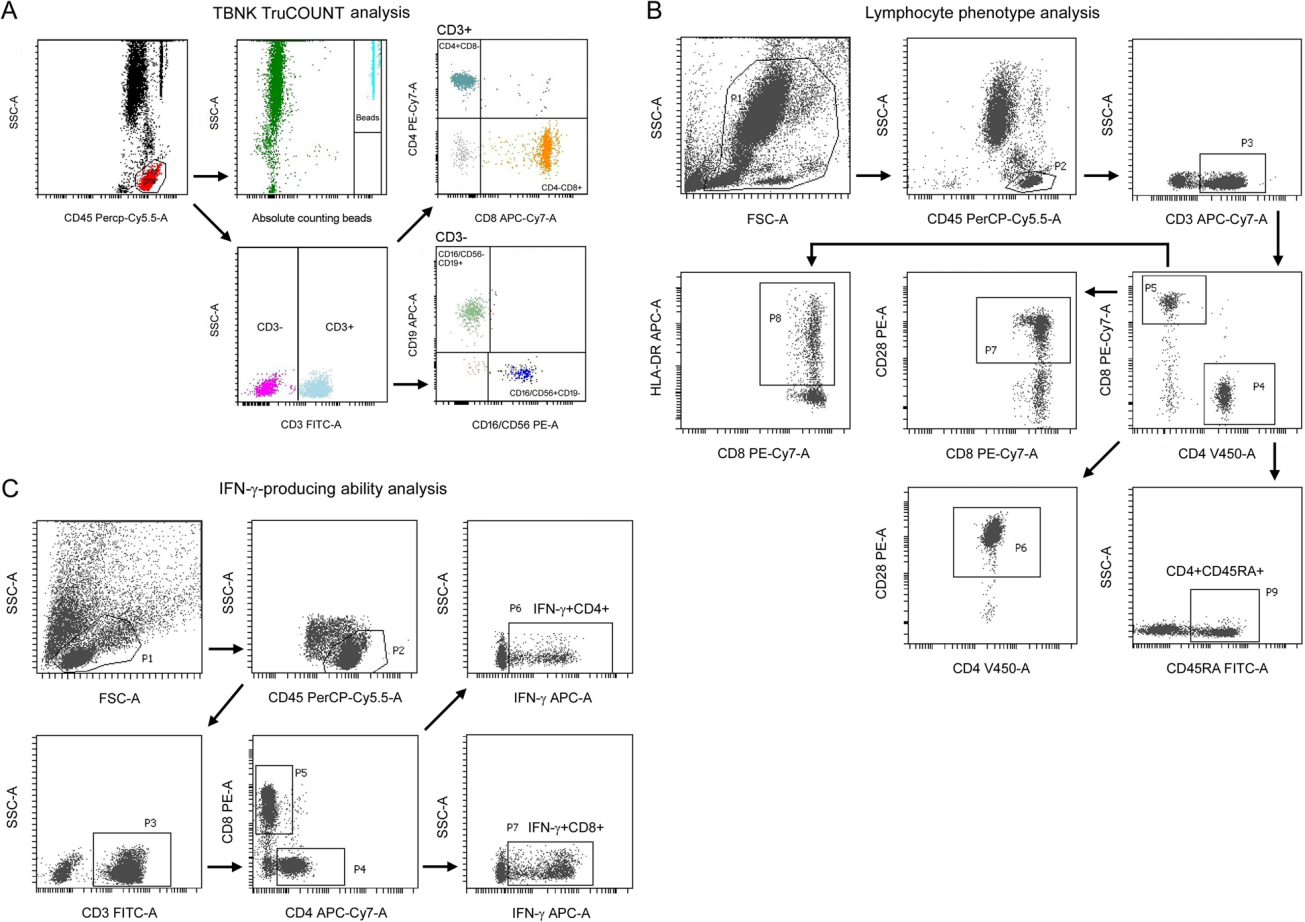
Gating strategies of lymphocyte parameter analysis. **A** Gating strategies of TBNK TruCOUNT analysis. **B** Gating strategies of lymphocyte phenotype analysis. **C** Gating strategies of intracellular IFN-γ production in CD4^+^ and CD8^+^ T cells after PMA/ionomycin stimulation

A volume of 2 mL heparinized venous blood was collected from each participant and processed by flow cytometry within 4 h. For lymphocyte subset counting, the percentages and absolute numbers of CD3^+^ T cells, CD3^−^CD19^+^ B cells, CD3^+^CD4^+^ T cells, CD3^+^CD8^+^ T cells and CD3^−^CD16^+^CD56^+^ NK cells were determined by using TruCOUNT tubes and BD Multitest 6-color TBNK Reagent Kit (BD Biosciences, San Jose, USA) according to the manufacturer's instructions. Briefly, 50 μL of whole blood was labeled with 6-color TBNK antibody cocktail for 15 min in room temperature. After adding 450 μL of FACS Lysing Solution, samples were analyzed with FACSCanto flow cytometer (BD Biosciences, San Jose, USA), using FACSCanto clinical software (BD Biosciences, San Jose, USA). Cells with CD45 high expression and with low side scatter were gated as lymphocytes. TruCOUNT beads were gated based on side scatter and fluorescence intensity. CD3^+^ cells in lymphocytes were gated as CD3^+^ T cells. CD4^+^CD8^−^ and CD8^+^CD4^−^ cells in CD3^+^ T cells were gated as CD3^+^CD4^+^ T cells and CD3^+^CD8^+^ T cells, respectively. CD19^+^CD16^−^CD56^−^ and CD16^+^CD56^+^CD19^−^ cells in CD3^−^ cells were gated as B cells and NK cells, respectively (Fig. 2A). For lymphocyte phenotype analysis, the following monoclonal antibodies were added to 100 μL of whole blood: anti-CD45 (catalog 652803), anti-CD3 (catalog 663490), anti-CD4 (catalog 560345), anti-CD8 (catalog 335822), anti-CD28 (catalog 662797), anti-HLA-DR (catalog 652809), and anti-CD45RA (catalog 662840) (BD Biosciences, San Jose, USA). Isotype controls with irrelevant specificities were included as negative controls. All of these cell suspensions were incubated for 20 min at room temperature. After lysing red blood cells, the cells were washed and resuspended in 200 μL of PBS. The cells were then analyzed with FACSCanto flow cytometer, using BD FACSDiva software (BD Biosciences, San Jose, USA). The gating strategies of CD28^+^CD4^+^ T cells, CD28^+^CD8^+^ T cells, CD45RA^+^CD4^+^ T cells, and HLA-DR^+^CD8^+^ T cells are shown in Fig. 2B.

For IFN-γ-producing ability analysis, phorbol 12-myristate 13-acetate (PMA)/ionomycin-stimulated intracellular IFN-γ production of lymphocytes was performed as described previously [15, 16]. The procedures are as follows: 1) 100 μL of whole blood was diluted with 400 μL of IMDM medium (Gibco-BRL, Grand Island, USA); 2) the diluted blood was incubated in the presence of Leukocyte Activation Cocktail (BD GolgiPlug™, including 50 ng/mL PMA, 1µM ionomycin and 1 µg/mL brefeldin A) for 4 h; 3) the cells were labeled with monoclonal antibodies (anti-CD45, anti-CD3, anti-CD4, and anti-CD8) (BD Biosciences, San Jose, USA); 4) the cells were fixed and permeabilized, then stained with intracellular anti-IFN-γ antibody (BD Pharmingen, San Diego, USA); and 5) the cells were analyzed with FACSCanto flow cytometer, using BD FACSDiva software. The gating strategies of IFN-γ^+^CD4^+^ T cells and IFN-γ^+^CD8^+^ T cells are shown in Fig. 2C.

To avoid the variation of flow cytometric analysis between laboratories, the following precautions were taken: 1) the standard protocol including sample preparation, staining procedure and gating strategy, was performed in all centers as described above, by using the same lot of reagents; 2) prior to sample analysis, mean fluorescence intensity of the CS&T Research Beads (BD Biosciences, San Jose, USA) of the same lot was used to set PMT voltages for each fluorescence channel, to reach the same mean fluorescence intensity on all FACSCanto cytometers in different centers; 3) the color compensation values were obtained by automatic compensation control system of the cytometer; 4) the same quality control samples (two levels) were performed in all centers each day during the experimental period, and the coefficients of variation were acceptable (high level sample < 10%; low level sample < 15%); and 5) all centers had passed the external quality assessment of TBNK lymphocyte counting conducted by Ministry of Health of China during the experimental period.

### Routine laboratory tests

The routine laboratory tests including routine blood test (white blood cell (WBC), neutrophil (NEUT), lymphocyte (LYMPH), monocyte (MONO), eosinophil (EOSIN), basophil (BASO), red blood cell (RBC), hemoglobin (HB) and platelet (PLT) etc.) and blood biochemistry (albumin (ALB), globulin (GLB), total cholesterol (TC), triglyceride (TG), high-density lipoprotein (HDL), low-density lipoprotein (LDL), and glucose (GLU) etc.) were performed with automatic analyzers (Sysmex XN9000, Mindray BC-6800Plus, Beckman-Coulter LH750, Beckman AU5800, Hitachi 7600 and Roche Cobas 701 etc.). All centers had passed the external quality assessment of these routine laboratory indicators conducted by Ministry of Health of China during the experimental period.

### Statistical analysis

The results of lymphocyte parameters were recorded and presented as mean ± standard deviation (SD) or median (25th and 75th percentiles), and the outliers were excluded from the analysis in each center. Continuous variables among different centers were compared with one-way ANOVA test, followed by Duncan's analysis for multiple comparison. Chi-square test was used for categorical data. Data with no statistical difference between multiple centers were pooled to establish the reference ranges of lymphocyte parameters. The pooled data were tested for normality by Kolmogorov–Smirnov test, and reference ranges were calculated using mean ± 1.96 SD for parametric data or 2.5% and 97.5% percentiles for non-parametric data.

Pearson correlation coefficient test was used to assess the relationship between two factors. Based on combined data from lymphocyte parameters and routine laboratory indicators, the regional difference among centers was determined by t-distributed stochastic neighbor embedding (t-SNE) analysis with R package “Rtsne”. Unsupervised hierarchical cluster analysis was performed to determine the relationships between lymphocyte parameters and routine indicators in healthy adults by using R package “pheatmap”, and represented as a dendrogram. Correlations among lymphocyte parameters or between lymphocyte parameters and routine laboratory indicators were analyzed and correlation matrix visualization was performed using the R package “corrplot”. Statistical significance was determined as *p* < 0.05. Statistical analyses were performed using SPSS version 19.0 (SPSS, Chicago, IL), GraphPad Prism 8.0 (San Diego, CA, USA), and R 4.0.3 (R Foundation, Vienna, Austria).

## Results

### Participants’ characteristics

After excluding individuals with outlier values of lymphocyte parameters from 10 centers, a total of 996 healthy adults fulfilled the inclusion criteria were included for analysis, including 485 (48.7%) males and 511 (51.3%) females. The mean age of the healthy adults was 40.07 years (SD, 12.58). The demographic, physiological, and routine laboratory characteristics of the total participants are shown in Table 1. The demographic, representative laboratory and lymphocyte parameter results of the participants at each center are shown in Table S1.

**Table 1:** The demographic, physiological, and routine laboratory characteristics of the participants

	**Healthy volunteers (*n* = 996)**
Age, years	40.07 ± 12.58
**Sex (male: female)**	485:511
**Han nationality**	996 (100%)
**Blood pressure**	
Systolic blood pressure (mmHg)	119.0 ± 13.51
Diastolic blood pressure (mmHg)	73.57 ± 9.443
**BMI**	22.70 ± 2.714
**Blood routine**	
WBC (× 10^9^/L)	5.950 ± 1.442
NEUT (× 10^9^/L)	3.417 ± 1.129
LYMPH (× 10^9^/L)	1.980 ± 0.538
MONO (× 10^9^/L)	0.396 ± 0.140
EOSIN (× 10^9^/L)	0.132 ± 0.103
BASO (× 10^9^/L)	0.025 ± 0.018
RBC (× 10^12^/L)	4.739 ± 0.487
HB (g/L)	142.0 ± 19.47
PLT (× 10^9^/L)	231.1 ± 55.79
**Biochemistry**	
ALB (g/L)	45.37 ± 4.077
GLB (g/L)	27.58 ± 3.983
TC (mmol/L)	4.394 ± 0.896
TG (mmol/L)	1.168 ± 0.553
HDL (mmol/L)	1.322 ± 0.315
LDL (mmol/L)	2.556 ± 0.649
GLU (mmol/L)	4.968 ± 0.722

Data are presented as mean ± SD, number (%), or otherwise indicated

*BMI* Body mass index, *WBC* White blood cell, *NEUT* Neutrophil, *LYMPH* Lymphocyte, *MONO* Monocyte, *EOSIN* Eosinophil, *BASO* Basophil, *RBC* Red blood cell, *HB* Hemoglobin, *PLT* Platelet, *ALB* Albumin, *GLB* Globulin, *TC* Total cholesterol, *TG* Triglyceride, *HDL* High density lipoprotein, *LDL* Low density lipoprotein, *GLU* Glucose

### Reference ranges of lymphocyte parameters

**Fig. 3:**
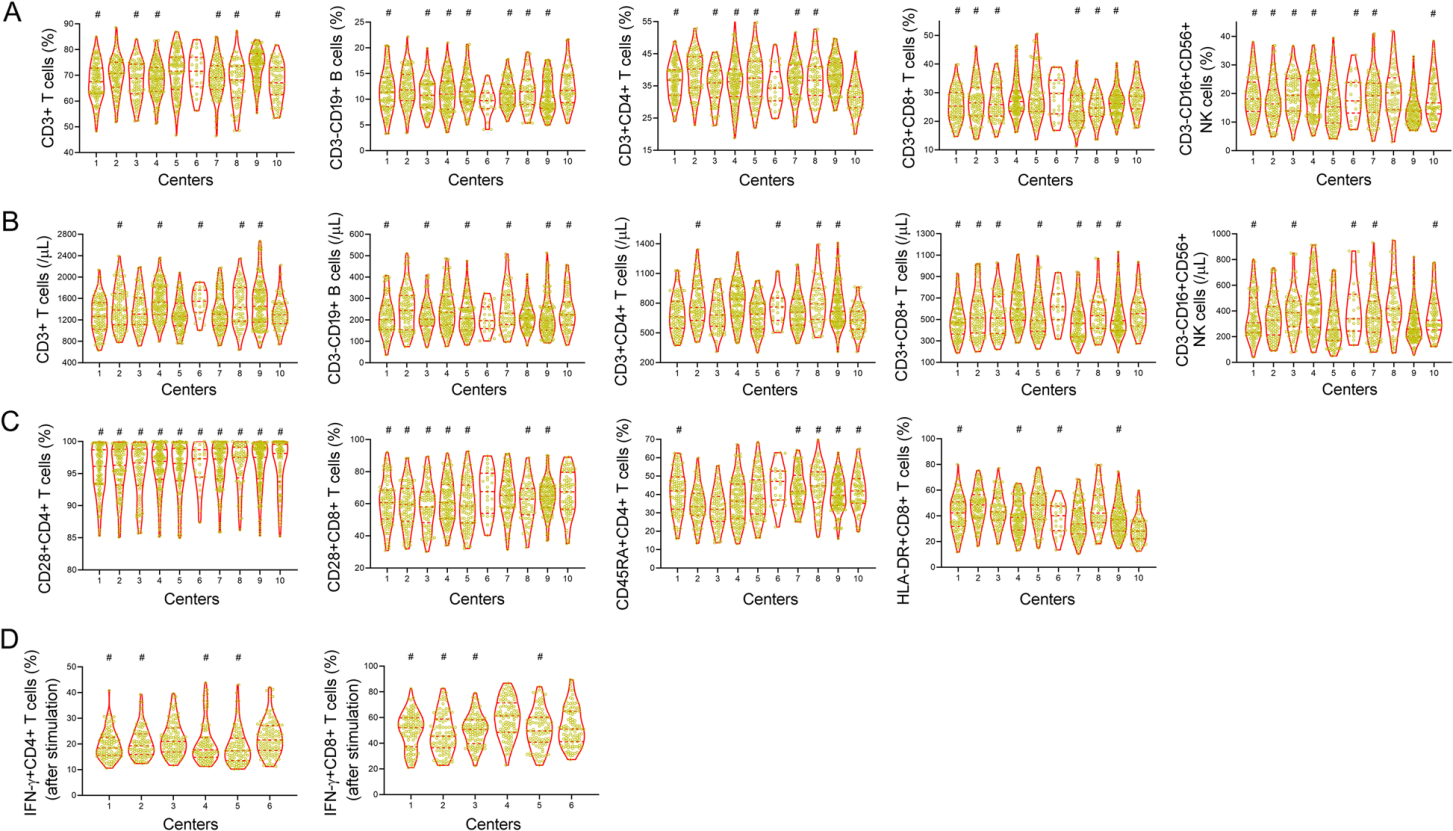
Lymphocyte parameter data in different centers. **A** Violin plots showing the results of lymphocyte subset percentages in 10 centers. **B** Violin plots showing the results of lymphocyte subset absolute numbers in 10 centers. **C** Violin plots showing the expression of surface markers CD28, CD45RA and HLA-DR on T cells in 10 centers. **D** Violin plots showing the results of intracellular IFN-γ production in CD4^+^ and CD8^+^ T cells after PMA/ionomycin stimulation in 6 centers. Data are presented as median and interquartile range (25th, 75th percentile). Each dot represents an individual donor. ^#^Data in these groups have no statistical significance

Due to the complexity of procedures, IFN-γ-producing ability analysis was only performed in 6 centers. Accordingly, the results of lymphocyte parameters, including the percentages and absolute numbers of lymphocytes (CD3^+^ T cells, CD3^+^CD4^+^ T cells, CD3^+^CD8^+^ T cells, CD3^−^CD19^+^ B cells, and CD3^−^CD16^+^CD56^+^ NK cells), the percentages of surface markers (CD28^+^CD4^+^ T cells, CD28^+^CD8^+^ T cells, CD45RA^+^CD4^+^ T cells, and HLA-DR^+^CD8^+^ T cells) in 10 centers, and the production of IFN-γ by CD4^+^ and CD8^+^ T cells after stimulation (IFN-γ^+^CD4^+^ T cells and IFN-γ^+^CD8^+^ T cells) in 6 centers, are shown in Fig. 3. Similar to routine laboratory indicators, almost all lymphocyte parameters showed a significant difference among different centers, except for CD28^+^CD4^+^ T cell percentage. Our results provide evidence that lymphocyte parameter data in different geographical areas of China show discrepancy to some extent.

A further Duncan test showed that lymphocyte parameter data from 4 to 10 centers had no statistical difference between each other. Hereafter, data in centers with no statistical difference were pooled to establish the reference ranges. As shown in Table 2, only a small part of lymphocyte parameters (CD3^+^CD4^+^ T cell percentage, CD28^+^CD8^+^ T cell percentage, CD45RA^+^CD4^+^ T cell percentage, HLA-DR^+^CD8^+^ T cell percentage, IFN-γ^+^CD8^+^ T cell percentage) passed normality test, and therefore mean ± 1.96 SD was used to define the normal ranges of these parameters. Except for these parameters, all the other lymphocyte parameters did not pass normality test and 2.5–97.5 percentiles were more suitable to define the normal ranges. Of these parameters only a few (CD3^+^ T cell percentage, CD3^+^CD4^+^ T cell percentage) exhibited a small coefficient of variation (CV) (approximately 10%), while many such as CD3^+^CD16^+^CD56^+^ NK cell number, CD28^+^CD4^+^ T cell percentage and CD3^+^CD19^+^ B cell number gave a CV over 30%, supporting the notion that lymphocyte parameter data are widely heterogeneous among healthy adults.

**Table 2:** Reference ranges of lymphocyte parameters in adults

Parameters	No.	Mean	Mean-1.96 SD to mean + 1.96 SD	Median	2.5 percentile to 97.5 percentile	CV
CD3^+^ T cells (%)	601	68.03	53.86-82.19	68.52	53.33-81.22	0.106
CD3^+^ T cells (/μL)	498	1494	754.8-2233	1464	876.8-2310	0.252
CD3^-^CD19^+^ B cells (%)	784	11.19	4.575-17.81	11.01	5.389-18.23	0.301
CD3^-^CD19^+^ B cells (/μL)	657	219.3	60.05-378.5	206	95.90-412.1	0.37
CD3^+^CD4^+^ T cells (%) ^a^	635	36.55	24.05-49.04	36.66	24.01-49.05	0.174
CD3^+^CD4^+^ T cells (/μL)	355	782.5	374.6-1190	752	455.7-1261	0.265
CD3^+^CD8^+^ T cells (%)	634	25.81	14.53-37.09	25.4	15.71-38.24	0.223
CD3^+^CD8^+^ T cells (/μL)	753	522.9	163.4-882.4	491	258.9-958.7	0.351
CD3^-^CD16^+^CD56^+^ NK cells (%)	664	18.88	4.554-33.21	18.37	6.369-34.83	0.387
CD3^-^CD16^+^CD56^+^ NK cells (/μL)	414	376.1	34.47-717.7	346	109.8-780.4	0.463
CD28^+^CD4^+^ T cells (%)	996	95.92	88.73-103.1	97	86.53-99.90	0.382
CD28^+^CD8^+^ T cells (%) ^a^	761	61.03	35.37-86.68	61.1	35.51-85.57	0.214
CD45RA^+^CD4^+^ T cells (%) ^a^	515	41.74	21.61-61.86	41.8	21.47-60.70	0.246
HLA-DR^+^CD8^+^ T cells (%) ^a^	423	39.42	13.49-65.35	38.29	17.36-64.08	0.335
IFN-γ^+^CD4^+^ T cells after stimulation (%)	396	19.79	7.081-32.49	18.15	11.50-36.96	0.327
IFN-γ^+^CD8^+^ T cells after stimulation (%) ^a^	405	49.39	21.96-76.81	49.6	24.88-78.87	0.283

*SD* Standard deviation, *CV* Coefficient of variation

^a^The data of this parameter pass normality test

### Cluster analysis of lymphocyte parameter data

**Fig. 4:**
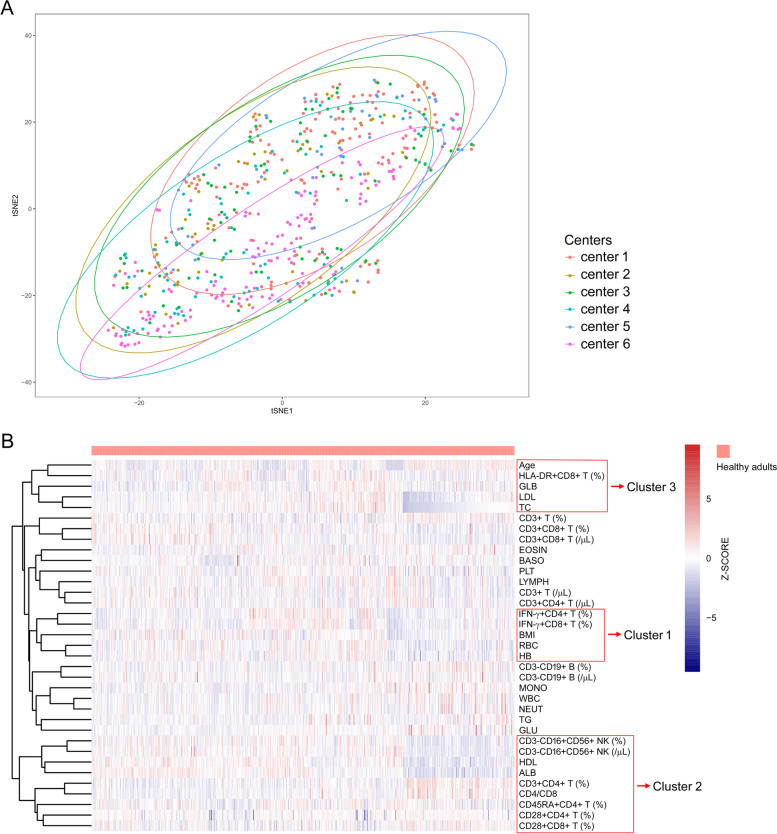
Cluster analysis of lymphocyte parameters. **A** The t-SNE analysis using 35 parameters (17 lymphocyte indicators: CD3^+^ T cell percentage, CD3^+^CD4^+^ T cell percentage, CD3^+^CD8^+^ T cell percentage, CD3^−^CD19^+^ B cell percentage, CD3^−^CD16^+^CD56^+^ NK cell percentage, CD3^+^ T cell number, CD3^+^CD4^+^ T cell number, CD3^+^CD8^+^ T cell number, CD3^−^CD19^+^ B cells number, CD3^−^CD16^+^CD56^+^ NK cell number, CD28^+^CD4^+^ T cell percentage, CD28^+^CD8^+^ T cell percentage, CD45RA^+^CD4^+^ T cell percentage, HLA-DR^+^CD8^+^ T cell percentage, IFN-γ^+^CD4^+^ T cell percentage, IFN-γ^+^CD8^+^ T cell percentage, and CD4/CD8; 18 routine parameters: age, BMI, WBC, NEUT, LYMPH, MONO, EOSIN, BASO,RBC, HB, PLT, ALB, GLB, TC, TG, HDL, LDL, and GLU) to clarify the regional difference of healthy adults among centers. **B** Hierarchical cluster analysis of lymphocyte and routine parameters together. The three red boxes represent 3 clusters. Dendrograms are shown as trees, representing the distance between variables. t-SNE, t-distributed stochastic neighbor embedding; BMI, body mass index; WBC, white blood cell; NEUT, neutrophil; LYMPH, lymphocyte; MONO, monocyte; EOSIN, eosinophil; BASO, basophil; RBC, red blood cell; HB, hemoglobin; PLT, platelet; ALB, albumin; GLB, globulin; TC, total cholesterol; TG, triglyceride; HDL, high density lipoprotein; LDL, low density lipoprotein; GLU, glucose

The t-SNE analysis was performed to compare the regional difference of healthy adults based on 35 parameters including 17 lymphocyte parameters and 18 routine laboratory parameters, and we observed that individuals in different areas were largely overlapped and could not be distinguished by these data (Fig. 4A). Of noted, a hierarchical cluster analysis of these 35 parameters found 3 interesting clusters which represented different immunological status. Cluster 1 represented the current lymphocyte function, which included lymphocyte parameters such as IFN-γ^+^CD4^+^ and IFN-γ^+^CD8^+^ T cells after stimulation. Notably, these current lymphocyte function parameters clustered together with routine indicators including body mass index (BMI), RBC and HB (Fig. 4B). Cluster 2 represented the potential of lymphocytes, which included lymphocyte parameters such as NK cell number, CD45RA^+^CD4^+^ T cell percentage, and CD28^+^CD8^+^ T cell percentage. Also interestingly, these lymphocyte potential parameters clustered together with routine indicators including ALB and HDL (Fig. 4B). Cluster 3 represented the inflammatory status, which included lymphocyte parameters such as HLA-DR^+^CD8^+^ T cell percentage, and we found inflammatory status parameter clustered together with routine indicators including LDL, TC, GLB and age (Fig. 4B). These data suggest that the common health indicators could be used to reflect different immunological status.

### Correlation analysis of lymphocyte parameters

**Fig. 5:**
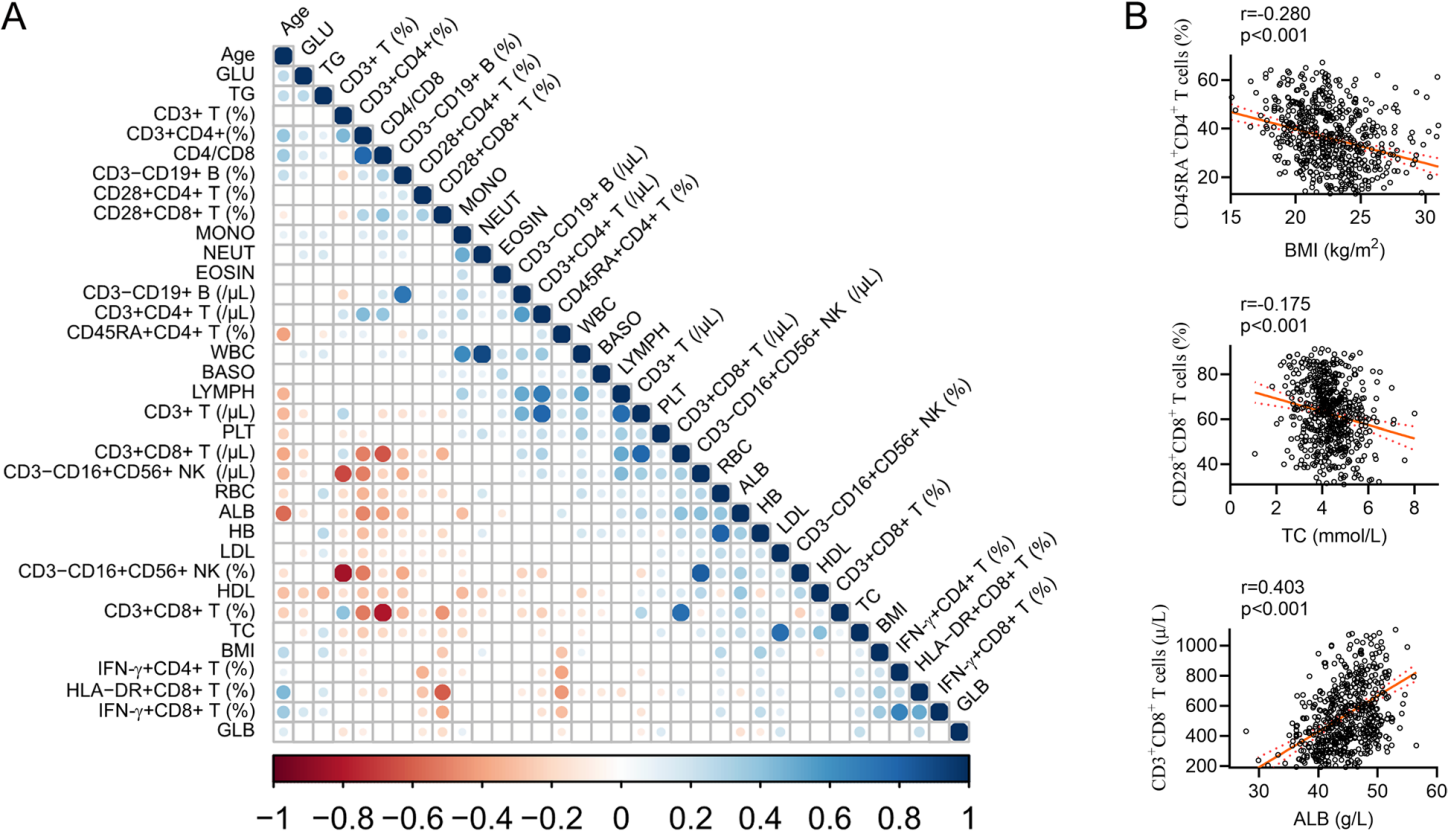
Correlation matrix analysis of lymphocyte parameters. **A** Pearson's correlation matrix analyzes all possible correlation between lymphocyte parameters and routine laboratory indicators. The correlation matrix shows the positive (blue) or negative (red) correlation of two parameters. Color intensity and circle size reflect correlation strength. Significance level was set at *p* < 0.05, and insignificance was shown as blank. **B** Representative dot pots showing the correlation between CD45RA^+^CD4^+^ T cell percentage and BMI, between CD3^+^CD8^+^ T cell number and ALB, and between CD28^+^CD8^+^ T cell percentage and TC. Each symbol represents an individual donor. BMI, body mass index; WBC, white blood cell; NEUT, neutrophil; LYMPH, lymphocyte; MONO, monocyte; EOSIN, eosinophil; BASO, basophil; RBC, red blood cell; HB, hemoglobin; PLT, platelet; ALB, albumin; GLB, globulin; TC, total cholesterol; TG, triglyceride; HDL, high density lipoprotein; LDL, low density lipoprotein; GLU, glucose

Finally, Pearson's correlation matrix was used to analyze all possible correlation coefficients between lymphocyte parameters and conventional laboratory indicators or between themselves. We summarized the representative correlations as follows. 1) Current lymphocyte function was negatively correlated with potential of lymphocytes, but positively with inflammatory status. It was clear that IFN-γ^+^CD8^+^ T cell percentage was significantly negatively correlated with CD28^+^CD8^+^ T cell percentage. Similarly, IFN-γ^+^CD4^+^ T cell percentage was significantly negatively correlated with CD45RA^+^CD4^+^ T cell percentage. 2) For nutritional indicators, ALB, RBC and HB were positively correlated with each other, and they were prominently positively correlated with lymphocyte potential parameters including CD8^+^ T cell number, NK cell number, and CD45RA^+^CD4^+^ T cell percentage. 3) For lipid profile, TC, TG and LDL were approximately positively correlated with nutritional indicators ALB, RBC and BMI, and they were positively correlated with current lymphocyte function parameters rather than lymphocyte potential parameters. Conversely, HDL was positively correlated with lymphocyte potential parameters including CD8^+^ T cell number and NK cell number. 4) For glucose metabolism, GLU was positively correlated with TG but negatively correlated with ALB. Notably, GLU was negatively correlated with lymphocyte potential parameters including CD3^+^, CD8^+^ T cell numbers and NK cell number. 5) For age, it was positively correlated with current lymphocyte function parameters such as IFN-γ^+^CD8^+^ T cell percentage and inflammatory status parameters such as HLA-DR^+^CD8^+^ T cell percentage, and also with GLU, TG, GLB and BMI, but negatively with lymphocyte potential parameters including CD4^+^ and CD8^+^ T cell number, NK cell number, and CD45RA^+^CD4^+^ T cell percentage (Fig. 5A). For instance, representative dot pots have shown the correlation between CD45RA^+^CD4^+^ T cell percentage and BMI, between CD28^+^CD8^+^ T cell percentage and TC, and between CD3^+^CD8^+^ T cell number and ALB (Fig. 5B).

## Discussion

In the present study, we successfully established the normal ranges of lymphocyte parameters in different areas. This study elucidates the key indicators used to reflect the current function or potential of lymphocytes, which may provide a valuable clue for how to keep immunity healthy. Given the influence of race, gender and age on peripheral lymphocyte composition is well-established, the use of domestic reference values has been widely emphasized to improve lymphocyte data interpretation [34]. In spite of this, the study still has some novelties. Firstly, this is the first multi-center study which includes Han population from most geographical areas of China. The study has tried to obtain the normal ranges of lymphocyte parameters which are suitable for use in different areas. Secondly, most previous studies focused on lymphocyte subset counting in different age and gender groups. Differently, to comprehensively understand lymphocyte status, the characteristics of lymphocyte parameters in the present study is surveyed from three aspects including the number, phenotype and cytokine-producing ability after stimulation. Thirdly, given that most individuals have performed lymphocyte parameter analysis and routine laboratory test simultaneously, we could rigorously and systematically analyze the relationship between lymphocyte parameters and common health indicators.

Theoretically, establishing the normal ranges of lymphocyte parameters across the country is convenient for data interpretation. However, the immunological parameter variation is related to many factors such as geographical location, age, and detection methods [27]. It is necessary to determine the variation of lymphocyte parameter ranges among different areas after excluding the influence of methodology. Accordingly, we conducted a 10-center study which included most areas of the country on Chinese healthy adults, by using the standard method. In line with the present findings [32], we confirmed that the variation of the normal ranges of most lymphocyte parameters was noted across the country. However, for some lymphocyte parameters like CD28^+^CD4^+^ T cell percentage, B or NK cell percentage, no or minor variation was observed among different areas and establishing the general normal ranges of these parameters might be practicable. In spite of the evidence of heterogeneity in lymphocyte parameter data among different geographical areas, the variation may actually be slight, as we could not be able to distinguish between centers even using all lymphocyte and routine laboratory parameters by t-distributed stochastic neighbor embedding analysis. This is in line with a recent study showing that no statistical significance was achieved in the number of lymphocytes from three regions of China [33].

In addition to the normal ranges of lymphocyte parameters, another aim of the study is to investigate the relationship between lymphocyte parameters and routine health indicators. It is known that the production of cytokine after stimulation can be used to reflect the current function of T cells, while the number of them, especially CD45RA^+^ naïve T cells, is the symbol of lymphocyte potential [16–18]. In addition, increased expression of HLA-DR on T cells contributes to persistence of chronic inflammation and correlates with severity of diseases [20–22]. Interestingly, we found that current lymphocyte function parameters (ie, IFN-γ^+^CD4^+^ T cell percentage and IFN-γ^+^CD8^+^ T cell percentage) clustered together with routine health indicators such as RBC, HB and BMI, whereas inflammatory status parameters (ie, HLA-DR^+^CD8^+^ T cell percentage) clustered together with TC and LDL. This is in line with the previous findings that the cytokine production of CD4^+^ T cells is more pronounced in obese individuals than in normal weight individuals [15]. Previous studies have also shown that RBC, HB and BMI are useful markers of malnutrition in adults [35, 36], and that host immunity is impaired in malnutrition people [15, 37]. This could be used to explain why IFN-γ^+^CD4^+^ T cell percentage and IFN-γ^+^CD8^+^ T cell percentage clustered together with RBC and HB. However, high level of lipid, contributing to high inflammatory status, may be one of the most crucial factors to cause cardiovascular disease [38]. Our findings support the benefit of high level of BMI as a symbol of strong lymphocyte function in the current state, but this correspondingly leads to the highly inflammatory environment of the host.

Just as a coin has two sides, high level of current lymphocyte function has its drawback. To be specific, high level of current lymphocyte function not only correlates with highly inflammatory status but also impairs the potential of lymphocytes, as the current function is obviously negatively correlated with lymphocyte potential. Consistent with this notion, IFN-γ-producing ability of CD4^+^ T cells is remarkably negatively correlated with CD45RA expression on them. Thus, although high level of BMI may increase the transient lymphocyte function, this would lead to reduced immune potential in the long run. As a result, maintaining the balance between current function and potential of lymphocytes is important to keep immunity healthy. In addition, increasing the number of T cells may be a better choice. Notably, the number of CD4^+^ T cells is not only positively correlated with CD45RA^+^CD4^+^ T cell percentage, but also with the number of other lymphocytes including CD8^+^ T cells, B cells, and NK cells. It means that expanding the number of lymphocytes, especially CD4^+^ T cells, may be the ideal way to keep healthy. Importantly, of the nutritional indicators only ALB is significantly positively correlated with CD45RA^+^CD4^+^ T cell percentage, which supports the key role of protein intake in maintaining healthy immunity through increasing both the number and the potential of lymphocytes. As expected, although lipid intake can promote current lymphocyte function, this reduces the potential of lymphocytes and harms to health in the long run.

Another interesting point is that our data emphasize the importance of age on lymphocyte parameters, which is in accordance with previous study showing that age-dependent immunological heterogeneity is greater than gender [28]. Previous studies have shown that except for some parameters such as NK cell number, CD28^+^CD8^+^ T cell percentage and HLA-DR^+^CD8^+^ T cell percentage, most lymphocyte parameters were comparable between genders [32]. In addition, even if these gender-related differences in lymphocyte parameters were statistically significant, it is generally viewed that the numeric differences between male and female were small and without clinical meaning [30]. We therefore did not classify the normal ranges of lymphocyte parameters by gender. Of note, we observed that age showed strong correlation with many routine laboratory and immunological indicators. Specifically, age was strongly positively correlated with TG, GLU, BMI, IFN-γ^+^CD8^+^ T cell percentage, HLA-DR^+^CD8^+^ T cell percentage, GLB and CD4/CD8, while negatively correlated with ALB, HDL, RBC, PLT, CD8^+^ T cell number, NK cell number and CD45RA^+^CD4^+^ T cell percentage. These data provide evidence that the increase of blood sugar and lipid, and body weight reduces the potential of immunity with increasing age throughout life.

Similarly, the impairment in both innate and adaptive immunity as a result of ageing is named immunosenescence, which is one of the hottest topics in recent decades [14]. Our data suggest that the decline of the number of lymphocytes is one of the typical features of immunosenescence. Besides, our findings are in line with previous reports which indicated the impaired potential of lymphocytes but with increased effector function in the elderly [39]. In addition, it is important to note that, the ratio of CD4^+^ to CD8^+^ T cells is also significantly increased with increasing age. Considering that the number of CD4^+^ T cells displays a decreased trend with increasing age (Figure S1), the loss of CD8^+^ T cells is extremely pronounced with increasing age and may be one of the most important factors to cause immunosenescence. Given that the number of CD8^+^ T cells is significantly positively correlated with ALB, RBC, HB, HDL and PLT but negatively correlated with GLU (Figure S2), we emphasize that the maintenance of ALB and RBC but avoiding an increase in GLU with increasing age may be important to keep longevity, through maintaining high level of CD8^+^ T cell number.

Several limitations should be mentioned. First, the presence of toxic habits such as smoking and alcohol consumption in healthy individuals was not taken into account, while these habits might affect the values ​​of lymphocytes in the peripheral blood. Second, given that lymphocyte parameter data in different geographical areas of China show discrepancy to some extent, the reference ranges of lymphocyte parameters established in this study may not be applicable in some areas. Third, due to the complexity of procedures, IFN-γ-producing ability analysis was not performed in all centers. Thus, the cluster and correlation analysis could not be conducted in all participants.

## Conclusion

Taken together, although the heterogeneity of lymphocyte parameters is widely found in different geographical areas of China, we have established the normal ranges of many lymphocyte parameters for healthy adults in different areas. Importantly, this study elucidates the relationship between lymphocyte parameters themselves as well as between lymphocyte parameters and routine nutritional indicators. Our findings emphasize the key role of ALB in maintaining both the current function and the potential of lymphocytes, which provides a clue for keeping immunity healthy.

## Supplementary Information

**Additional file 1: Supplementary Table 1.** The demographic, representative laboratory, and lymphocyte parameter results of the participants from 10 centers. **Supplementary Figure 1. **Correlation between CD3^+^CD4^+^ T cell number and age. Each symbol represents an individual donor. **Supplementary Figure 2. **Correlation between CD3^+^CD8^+^ T cell number and other routine indicators including RBC, HB, HDL, PLT, and GLU. Each symbol represents an individual donor. RBC, red blood cell; HB, hemoglobin; HDL, high density lipoprotein; PLT, platelet; GLU, glucose.

## Data Availability

All data is included in the manuscript and/or supporting information.

## Abbreviations

NK: Natural killer
WBC: White blood cell
NEUT: Neutrophil
LYMPH: Lymphocyte
MONO: Monocyte
EOSIN: Eosinophil
BASO: Basophil
RBC: Red blood cell
HB: Hemoglobin
PLT: Platelet
BMI: Body mass index
ALB: Albumin
GLB: Globulin
TC: Total cholesterol
TG: Triglyceride
HDL: High-density lipoprotein
LDL: Low-density lipoprotein
GLU: Glucose
SD: Standard deviation
t-SNE: T-distributed stochastic neighbor embedding
CV: Coefficient of variation
