# Supplementary material for: The reference ranges and characteristics of lymphocyte parameters and the correlation between lymphocyte parameters and routine health indicators in adults from China
Source: Immun Ageing. 2022 Sep 27;19:42. doi: 10.1186/s12979-022-00298-5 (PMC9513899; doi:10.1186/s12979-022-00298-5)
Supplement: Supplementary file 1 — Additional file 1: Supplementary Table 1. The demographic, representative laboratory, and lymphocyte parameter results of the participants from 10 centers. Supplementary Figure 1. Correlation between CD3+CD4+ T cell number and age. Each symbol represents an individual donor. Supplementary Figure 2. Correlation between CD3+CD8+ T cell number and other routine indicators including RBC, HB, HDL, PLT, and GLU. Each symbol represents an individual donor. RBC, red blood cell; HB, hemoglobin; HDL, high density lipoprotein; PLT, platelet; GLU, glucose. [file 12979_2022_298_MOESM1_ESM.pdf]

Supplementary Table 1. The demographic, representative laboratory, and lymphocyte parameter results of the participants from 10 centers

	Center 1 (n=106)	Center 2 (n=108)	Center 3 (n=87)	Center 4 (n=141)	Center 5 (n=114)	Center 6 (n=25)	Center 7 (n=118)	Center 8 (n=70)	Center 9 (n=143)	Center 10 (n=84)	*p* value
Age, years	38.52 ± 11.06	39.86 ± 10.56	33.86 ± 12.38	42.35 ± 13.93	48.65 ± 10.30	39.60 ± 10.43	37.75 ± 11.12	39.67 ± 10.20	39.06 ± 13.04	38.65 ± 14.83	<0.001
Sex (male : female)	56:50	43:65	34:53	64:77	55:59	8:17	72:46	34:36	71:72	48:36	
**Laboratory results**											
WBC ($\times10^9$/L)	6.160 ± 1.105	5.662 ± 1.273	5.717 ± 1.013	5.936 ± 1.191	5.639 ± 1.157	5.910 ± 1.073	6.316 ± 1.382	6.162 ± 1.312	6.112 ± 1.671	6.322 ± 1.305	<0.001
NEUT ($\times10^9$/L)	3.596 ± 0.946	3.254 ± 0.991	3.253 ± 0.787	3.382 ± 0.896	3.289 ± 0.896	3.181 ± 0.723	3.666 ± 1.070	3.551 ± 0.968	3.614 ± 1.366	3.661 ± 1.134	<0.001
LYMPH ($\times10^9$/L)	2.054 ± 0.451	1.913 ± 0.425	1.988 ± 0.442	1.992 ± 0.464	1.851 ± 0.410	2.173 ± 0.486	2.055 ± 0.581	2.035 ± 0.457	1.789 ± 0.540	2.121 ± 0.516	<0.001
MONO ($\times10^9$/L)	0.353 ± 0.096	0.364 ± 0.099	0.345 ± 0.107	0.395 ± 0.145	0.357 ± 0.108	0.361 ± 0.079	0.416 ± 0.114	0.434 ± 0.126	0.428 ± 0.118	0.397 ± 0.102	<0.001
RBC ($\times10^{12}$/L)	4.708 ± 0.446	4.738 ± 0.418	4.584 ± 0.613	4.756 ± 0.461	4.755 ± 0.412	4.592 ± 0.363	4.997 ± 0.486	4.822 ± 0.415	4.436 ± 0.604	4.856 ± 0.579	<0.001
**parameters**											
$CD3^+$ T cells (%)	67.55 (62.88-72.84)	70.8 (65.33-75.02)	68.84 (62.82-74.35)	68.57 (63.81-73.03)	71.59 (64.55-77.02)	71.59 (65.54-77.02)	69.13 (64.52-73.74)	68.21 (61.28-73.63)	74.69 (69.18-78.46)	67.08 (62.48-73.03)	<0.001
$CD3^+$ T cells (/μL)	1265 (1027-1523)	1384 (1109-1696)	1307 (1111-1612)	1532 (1299-1815)	1267 (1091-1510)	1549 (1340-1756)	1304 (1096-1603)	1434 (1175-1805)	1451 (1160-1762)	1296 (1133-1496)	<0.001
$CD3^-CD19^+$ B cells (%)	11.38 (8.538-14.23)	11.81 (9.74-14.82)	10.76 (8.435-12.98)	10.99 (8.04-13.52)	11.45 (9.48-13.77)	9.73 (8.155-11.23)	11.07 (8.895-12.85)	11.46 (8.97-13.88)	10.4 (8.193-13.55)	11.67 (9.35-14.65)	<0.001
$CD3^-CD19^+$ B cells (/μL)	202 (155.3-268)	244.5 (153.8-314.5)	202.5 (171.5-257.8)	236 (175.8-309.8)	205.5 (153.5-257.8)	197 (159.5-261)	231 (178.5-316.5)	209 (165.8-248)	199.5 (152-261.3)	224 (177-284)	<0.001
$CD3^+CD4^+$ T cells (%)	36.81 (32.08-40.05)	40.26 (34.37-44.03)	35.94 (31.39-39.74)	36.3 (31.71-40.22)	37.38 (31.66-42.66)	34.31 (30.34-39.46)	37.43 (32.38-41.48)	36.75 (32.03-41.05)	38.34 (34.84-42.2)	31.47 (28.14-34.98)	<0.001
$CD3^+CD4^+$ T cells (/μL)	663 (547-817.5)	756.5 (625.8-948.8)	680 (565.5-828.5)	839 (669-971)	689 (539-798.5)	761 (625.5-851)	709 (577.8-840.3)	793 (635.5-950.5)	719 (613.5-884.3)	630 (536-719)	<0.001
$CD3^+CD8^+$ T cells (%)	25.3 (21.45-30.01)	26.42 (21.83-31.82)	25.79 (21.82-31.8)	26.91 (23.53-30.64)	27.7 (23.45-35.24)	29.73 (22.58-34.39)	23.6 (20.15-29.09)	24.61 (21.68-27.97)	26.2 (22.77-29.01)	28.66 (24.34-31.67)	<0.001
$CD3^+CD8^+$ T cells (/μL)	470 (364-597.3)	514.5 (373.8-672.5)	513.5 (369-714.3)	599 (493-755)	491.5 (390-658.8)	618 (498.5-740.5)	464 (337.8-574.8)	535 (416-662)	489 (394.8-630.5)	553 (439-657)	<0.001
$CD3^-CD16^+CD56^+$ NK cells (%)	18.13 (13.7-23.98)	16.37 (12.14-21.35)	19.4 (13.85-25.23)	19.45 (12.54-24.61)	15.28 (9.68-21.4)	17.42 (13.17-23.84)	19.23 (13.38-23.66)	20.24 (14.34-25.51)	13.85 (10.35-17.32)	16.71 (12.86-23.53)	<0.001
$CD3^-CD16^+CD56^+$ NK cells (/μL)	308 (230.8-502.3)	328.5 (213.8-433.8)	385.5 (278.5-478.3)	449 (272.5-607)	253 (170.5-405)	339 (244-530)	344.5 (234-476.3)	418 (314-579)	257.5 (199-381)	328 (253-458)	<0.001
$CD28^+CD4^+$ T cells (%)	96.12 (93.39-98.7)	96.3 (93.2-98.78)	96.65 (93.13-98.83)	96.9 (94.1-98.7)	96.55 (93.88-98.9)	97.3 (94.45-98.7)	97.25 (94.18-98.93)	97.5 (94.32-99.1)	97.5 (94.22-99.02)	98.13 (93.7-99.57)	0.432
$CD28^+CD8^+$ T cells (%)	60.74 (50.58-68.48)	59.5 (49.1-69.6)	58 (48.2-67.45)	60.7 (50.8-71.9)	58.55 (48.08-71.63)	67.6 (54-79)	65.2 (57.5-74.25)	62.89 (53.18-69.53)	64.72 (57.62-70.8)	67.41 (56.64-79.59)	<0.001
$CD45RA^+CD4^+$T cells (%)	41.98 (32.02-49.56)	33.4 (28.6-40.6)	31.9 (25.4-38.95)	36.3 (26.88-45.7)	37.7 (29.4-47.98)	47.1 (37-52.75)	41.8 (34.85-50.4)	44.7 (35.75-52.3)	39.53 (34.06-46.68)	41.91 (35.22-48.6)	<0.001
$HLA\text{-}DR^+CD8^+$ T cells (%)	42.3 (31.74-50.93)	48.55 (38.5-56.5)	43.1 (36.65-53.78)	38.8 (28.83-51.25)	48.35 (37.25-57.28)	40.1 (28.5-47.6)	33.8 (26-45.83)	42.1 (34.6-56.17)	36.95 (28.24-46.15)	28.07 (22.13-35.61)	<0.001

Data are presented as mean ± SD or median (25th-75th). WBC, white blood cell; NEUT, neutrophil; LYMPH, lymphocyte; MONO, monocyte; RBC, red blood cell. Multiple comparison was performed by one-way ANOVA.

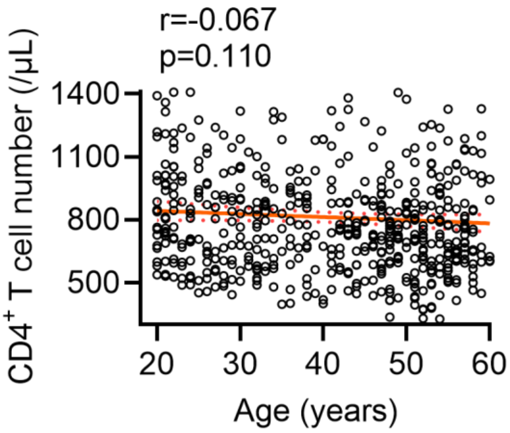

Supplementary Figure 1 Correlation between $CD3^+CD4^+$ T cell number and age. Each symbol represents an individual donor.

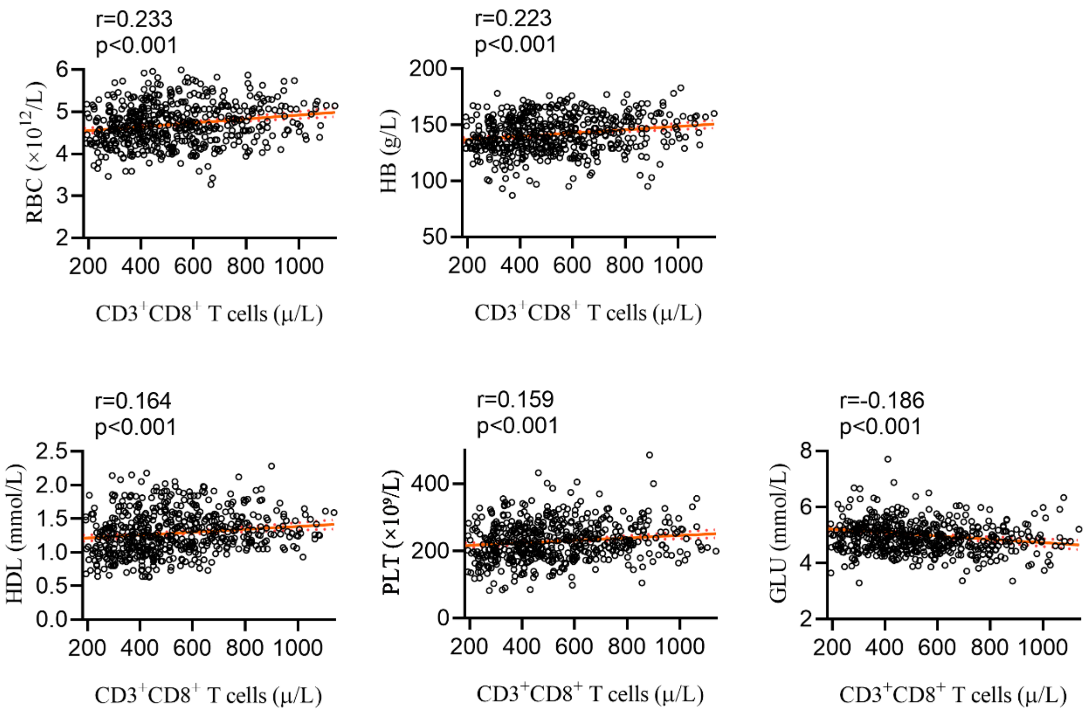

Supplementary Figure 2 Correlation between $CD3^{+}CD8^{+}$ T cell number and other routine indicators including RBC, HB, HDL, PLT, and GLU. Each symbol represents an individual donor.
